# Back-Health Knowledge and Misconceptions Related to the Daily Life Activities of Secondary School Students

**DOI:** 10.3390/children11080997

**Published:** 2024-08-15

**Authors:** Manuel Monfort-Pañego, Antonio Hans Bosch-Biviá, Vicente Miñana-Signes, Matias Noll

**Affiliations:** 1 Physical Education Teacher Education Department, Teacher Education Faculty, University of Valencia, 46022 Valencia, Spain; antonio.bosch@uv.es (A.H.B.-B.); vicente.minana@uv.es (V.M.-S.); 2 Nutrition Faculty, Universidade Federal de Goiás, Goiânia 74000, Brazil; matias.noll@ifgoiano.edu.br

**Keywords:** concept-based physical education, learning, prior knowledge, naïve conception, conceptual change

## Abstract

High school students with better knowledge about back care have fewer problems, but conceptual errors can hinder the acquisition of essential knowledge necessary for developing healthy habits. This study analyzed secondary school students’ declarative knowledge and misconceptions related to back care in daily activities. An exploratory cross-sectional study was conducted with 80 girls and 89 boys aged 14–18 years (M = 15.68, SD = 2.12). The Health Questionnaire on Back Care Knowledge in Activities of Daily Living was used to evaluate knowledge using the true answer model (TAM) and the misconception model (MM). Using the test–retest method, both models’ reliability was confirmed (TAM = 0.75; MM = 0.77), while only a minimal measurement error was identified (TAM = −0.01; MM = −0.07). The average scores were 6.23 for the TAM and 2.29 for the MM. The results showed no significant differences in both models. The analysis indicated that students had the most accurate knowledge of the location and function of the spine, whereas misconceptions regarding anatomical understanding and body posture usage were common. An analysis of the results under Reassumption Theory emphasizes the significance of comprehending concepts such as load transmission and spinal stability to maintain back health, thus highlighting the need for improved education in these areas to address misconceptions and enhance overall back-care knowledge.

## 1. Introduction

Currently, musculoskeletal health is a major health concern worldwide [1]. Meanwhile, epidemiological studies indicate that back pain is one of the most prevalent pathologies in developed societies [2]. Further, nonspecific lower back pain [3] and its associated psychosocial factors [4] are the leading causes of work incapacity [1] and have a significant impact on individuals’ health and quality of life.

Physical activity (PA) is recognized as one of the factors with the greatest medium- and long-term impacts on health [5]. Research on this topic has mainly focused on PA’s effects on diseases with high morbidity rates, such as coronary heart disease, obesity-related diseases [6], and cancer [5]; however, scarce attention has been paid to its effects on the musculoskeletal system.

Physical education (PE), as a school subject, has aligned with these trends, while morbidity problems and the prevention perspective have been integrated into the official curricula of all developed countries. Research has examined the development of PE to improve the physical, affective, social, and cognitive domains and has assessed lifestyle factors such as the time and intensity of PA [7]. Although the study of spinal pathologies is a topic of great interest in the biomedical field, back health (from the social and educational perspectives) remains notably underrepresented in international research [8]. The role of body awareness and posture, quality in the execution of movements, and the level of knowledge about the practices of quality are greatly overlooked in the study of healthy PA. Consequently, there is a notable gap in our understanding of back-health education’s effect on overall health [9].

To advance health education research, it is necessary to adopt new perspectives that offer a more comprehensive understanding. Studies on PA and health [10] can serve as an important reference point from which to address the inclusion of back health in health education programs and educational interventions. One of the new lines of research in this area is to determine knowledge’s role in achieving this goal [11].

### 1.1. Knowledge’s Role in the Development of a Healthy Lifestyle

Studies analyzing the importance of health-related PA knowledge in PE lessons for the acquisition of active lifestyle habits use two main arguments [12]. First, they are based on a behavioral skills model of informing and motivating [13]. It seems logical to assume that to develop healthy active daily life habits autonomously, it is necessary to have sufficient knowledge of PA practices’ effects on health. Knowing what PAs to do and how to do them in a healthy way is necessary to acquire and maintain favorable habits. This knowledge is necessary not only to perform self-regulated PA but also to maintain motivation and develop behaviors to ensure that PA is established and maintained [13]. Some authors have hypothesized that dropout from PA in adulthood is higher if there is insufficient knowledge about the importance of exercise in relation to health [14]. The second line of argument is that knowledge is important as a prerequisite for the acquisition of habits or changes in healthy behavior in young people [15]. Important reviews on the effectiveness of health interventions [13] or increases in PA [16] point to the positive effect of knowledge interventions, leading to increased PA [12]. However, it can be said that knowledge alone is unlikely to exert a behavior-change effect [17], although research on the mediators between PA and habit acquisition is limited [18]. From the perspective of the behavioral skills model, it has been suggested that knowledge’s effect on habits is mediated by behavioral skills [13].

However, few studies have attempted to specifically analyze knowledge’s role in PA practice through a correlational approach [12], while most have done so in combination with other factors [17]. Thus, it has been shown that adolescents acquiring the active lifestyle habits recommended by different international organizations (the World Health Organization, the Society of Health and Physical Education, and the National Institute for Health and Clinical Excellence) have higher health-related PA knowledge levels compared with sedentary adolescents [14]. Similarly, studies based on the theoretical conceptualization of motor literacy have shown that students in educational interventions that have implemented holistic models have achieved higher levels of PA than those using more traditional methods [19]. Despite the evidence from studies on specific knowledge’s positive effect on PA and fitness, the lack of causal studies [12,20] that explain in depth the role of knowledge in the acquisition of appropriate habits means that, currently, it can only be said that knowledge is a prerequisite for—but not a guarantee of—the acquisition and maintenance of healthy habits. The link between knowledge and changes in people’s engagement in physical activities seems to be missing.

### 1.2. What We Mean by Knowledge

Although knowledge’s role in the acquisition of habits remains to be determined, it is important to delimit the concepts used in this study. From a broader perspective, Polanyi [21] introduced the concepts of tacit and explicit knowledge, which were included in the model proposed by Alexander et al. [22]. This model structures knowledge as tacit, procedural, explicit, and declarative. Procedural knowledge refers to “knowing how”, i.e., the skills and procedures that we have learned and can perform, often without needing to think consciously about the individual steps involved in doing so. Declarative knowledge refers to “knowing what”, i.e., the facts and information that a person can verbally state. Both are interconnected and present in the learning process. According to Nonaka [23], to enhance the quality of individuals’ knowledge, the “knowing how” has to be subjected to a constant interplay with the evolution of “knowing what.”

### 1.3. Knowledge as a Barrier or Impetus to Understanding: Reassumption Theory

Investigating students’ knowledge is not only about determining what they know in relation to standard knowledge or models. In line with constructivist theories, the theory of reassumption [24], one of the multiple routes to successful learning [25], explains that knowledge and beliefs are assumed in a continuous and routine manner, extending existing knowledge and beliefs from the same perspective or theory without revising them. For a change to occur in this assumption of knowledge, there must be a situation in which a phenomenon understood from one theory is contrasted with another new theory (bisociation). When this knowledge is also understood from another theory and enters into competition or conflict with the first, this is the moment when the possibility of change is sown. It is from this conflict, in which the understanding of the concept is finally seen with greater clarity and coherence from the perspective of the new theory, that conceptual change takes place. Studies have shown that students’ prior knowledge (understood as knowledge acquired unconsciously through use and preceding a learning process) and misconceptions (understood as knowledge that is built on assumptions that do not correspond to the truth) can aid or hinder the acquisition of new concepts, or even restructure existing ones [26]. When prior knowledge of a concept related to a topic is deeply ingrained and in conflict with new information, it can become a problem for knowledge acquisition, thereby creating resistance to conceptual change [27]. Being aware of prior knowledge and misconceptions, or the most common errors of comprehension in learning, is essential for teachers [28].

### 1.4. Declarative Knowledge, Prior Knowledge, and Misconception in Back Health

However, research on declarative knowledge in the PE field is scarce [29]. Although some research has examined topics of practical origin in teaching the motor actions of sports or understanding tactical play [30], research on conceptual errors in declarative or theoretical knowledge has been marginal, with some studies focusing on concepts such as energy expenditure [31].

In the field of health and back care, some of the most important concepts are spine stability and the functioning of the trunk musculature’s mechanical, physiological mechanisms for its preservation, and the role of different fitness factors in back health, such as flexibility. [32,33,34]. The literature has addressed the problem of the declarative knowledge on back health. Studies have shown that secondary school students have insufficient knowledge and that students with better knowledge have a lower incidence of back pain [9]. However, nothing is known about conceptual errors that could affect the correct acquisition of new concepts necessary for the development of adequate healthy habits.

Given the lack of studies on students’ knowledge concerning back care in daily life activities, our study aimed to analyze declarative knowledge about back care in daily life activities to reveal and explain the prior knowledge and misconceptions of secondary school students.

## 2. Materials and Methods

A non-randomized, exploratory, cross-sectional study was designed using an initial sample of 183 students from three secondary schools in València, Spain. Two schools were selected based on their accessibility. The final sample consisted of 80 girls and 89 boys aged 14–18 (M = 15.68, SD = 2.12). There was a loss rate of 7.6%. The margin of error for this sample was ±3.3. All the students in the natural classroom group were included in this study. Participants with a percentage of unanswered questions ≥ 5 were not included in the study. The data collection protocols, data protection procedures, parental consent forms, and student assent forms were approved by the university’s ethics committee (H1509086047576).

The validated Health Questionnaire on Back Care Knowledge in Daily Living Activities (HEBACKNOW) was used to examine students’ declarative knowledge [8]. The questionnaire was administered during PE classes in the school computer room using Google Forms. Each test had a mean response time of 12.26 min. This instrument included 24 multiple-choice questions on basic (seven items) and applied (17 items) knowledge. The questions were grouped into seven knowledge categories: topographical-anatomical, functional, standing posture, sitting posture, lying posture, backpack carrying, and heavy lifting. The questionnaire was scored in its first version, where each correct answer was scored as one point; this was called the true answer model (TAM). A second scoring method was used to determine the errors in the answers; this was called the misconceptions model (MM). Incorrect answers were scored as one point. The final scoring scale was 0–10 points for each category and for the total, and it was averaged per category and total in each model.

The internal consistency of the questionnaire was analyzed to check the scoring models of the instrument. The reliability of the instrument scores was tested using a test–retest design with two sets of data collected two weeks apart. The values of each response according to the models are listed in Supplementary Materials (Tables S1–S12, Supplementary File S1).

The technical verification of the correct functioning of the measuring instrument used in this study was complex and extensive. However, as this was not the main aim of the study, it was decided to transfer this entire process to Supplementary Files S1 and S2. This process aimed to make the results more consistent, thus guaranteeing the accuracy of the study. Simultaneously, it made the report easier to read, emphasized the main purpose of the study, and made all information more accessible.

The means of the first round were used to examine the results of the different models. The frequencies of correct and incorrect answers were analyzed to identify the best-known concepts and the most common misconceptions (Tables S1–S12, Supplementary File S1).

## 3. Results

The main results of this study showed that the HEBACKNOW was reliable for both the collection of information on the TAM and the study of MM in secondary school students. Both the correlation statistics (Figures S1 and S2, Supplementary File S2) and repeated-measurement error statistics (Tables S13 and S14, Supplementary File S2) performed well. Plotting the mean scores of both administrations and calculating the slope of their linear functions in all scoring models showed a positive relationship between the measurements at the different timepoints, while the difference in the measurements was always <5% (Figures S3–S6, Supplementary File S2).

The regression analysis of the means of the total scores and their differences for the TAM (β = 0.18; 95% CI [0.02, 0.32]; t_168_ = 2.17; *p* = 0.03; R^2^ = 0.03; adjusted R^2^ = 2.2%) and MM (β = 0.17; 95% CI [0.02, 0.31]; t_168_ = 2.27; *p* = 0.03; R^2^ = 0.03; adjusted R^2^ = 2.4%) indicated a significant increase in score differences as mean scores increased, albeit with a low probability of prediction (<3%).

No floor/ceiling effect was observed (item difficulty), as scores within the 0–1 and 9–10 range had a frequency of 0%. The discriminatory power of the instrument’s total score was demonstrated (Supplementary File S2). This balanced instrument was not influenced by gender or age (Supplementary File S2).

Descriptive results for the items are presented in Supplementary File S1. A summary of the percentages of hits and misses for each question is presented in Table 1. The TAM results (Table S13, Supplementary File S2) indicated that, in general, the study population achieved an average level of knowledge (6.23) concerning the use of the body in daily life for the development of a healthy spine. The highest level of knowledge was for anatomical–functional knowledge, followed by knowledge of weight load and sitting posture. There were five items for which the percentage of correct answers was over 80% (Table 1): the location of the spine (V1), naming the different parts (V3), the function of the spine in the body (V5), the function of the trunk muscles (V7), and how to carry weight in bags (V18). There were three items with percentages below 50%: knowing the name of the trunk musculature (V6), knowing the most stressful posture for the back (V8), knowing the correct posture while sitting at a desk (V12).

The results of the MM (Table S14, Supplementary File S2) showed that the study sample had an average of more than two errors per participant (2.29). The categories with the highest percentage of errors were anatomical knowledge and use of standing posture, which exceeded the average of three errors per participant. The items for which more than 50% of participants made errors (Table 1) concerned the number of back curves (V2) and knowledge of the most stressful posture for the back (V8). The item regarding the correct sitting posture in front of a desk (V12) exhibited more than 30% errors, while the item regarding the most appropriate back posture when standing (V9) exhibited more than 20% errors. Another nine items exhibited a > 10% error rate (V6, V11, V15, V18, V19, V21, V22, V23, V24), and four items (V3, V7, V10, V14) exhibited a > 5% error rate. The rest exhibited <5% incorrect answers.

## 4. Discussion

### 4.1. Reliability of the Results in TAM and MM

First, the reliable scores of the instrument in the study sample (Table S13) allowed us to analyze the participants’ level of correct prior knowledge [8,35]. Second, this study showed, for the first time, the reliable use of incorrect answers to assess misconceptions (Table S14). This knowledge allowed us to determine the gaps in the understanding of fundamental concepts, thus facilitating deep learning in the development of solid knowledge for use in everyday activities (Table 1).

In our view, understanding both the correct prior knowledge and misconceptions of students when teaching healthy habits for back care can greatly enhance teachers’ effectiveness [36]. By providing comprehensive information, teachers can help students identify areas for improvement and make informed decisions about autonomously and responsibly using their bodies in daily activities. This approach to acquiring new habits or modifying existing ones has a significant impact on students.

### 4.2. Prior Knowledge and Misconceptions in Back-Care Learning

The results of this study highlight the complexity of how adolescents acquire basic concepts related to back health and how their knowledge acquisition and use may reveal learning problems. Overall, the results indicated that declarative knowledge of back care in daily life activities was sufficient (6.23 points). However, this knowledge was related to the basic activities of daily life. Thus, the correct understanding of one concept should allow participants to correctly respond to the other items; therefore, the expected score is >80%. From this point of view, the scores obtained are far from the adequate declarative knowledge necessary for good use of the body in activities of daily living.

The most consolidated declarative knowledge above the average score was related to the location of the spine (Table S1, variable V1), its function (Table S3, variable V5), and trunk musculature (Table S3, variable V6), as well as the balanced carrying of weight in bags with both hands (Table S9, variable V18). Topographical knowledge, as well as a balanced distribution of weight on both arms, is not as much complex knowledge as it is common sense. However, knowledge of the functions of the spine and trunk musculature are items that address more complex knowledge and have important implications for understanding the use of the body in different postures. From the current scientific knowledge [37], the concepts of load or weight support and transmission, along with trunk stability, are linked to the understanding of the mechanical and neurophysiological mechanisms involved in the performance of body movements or posture holding [38]. A thorough understanding of these mechanisms is key to the correct analysis of how we should use our bodies to apply this knowledge to different situations in the future. When concepts are not well understood and consolidated, this leads to the use of naïve concepts or misconceptions regarding how to use the body in different situations.

Based on our results regarding misconception knowledge, it seems logical to assume that if the categories of anatomical knowledge and lying posture were the lowest-scoring categories in the TAM, they would be the highest-scoring categories in the MM. However, in our results, this assumption was similar to that based on anatomical knowledge. This is explained by the fact that, in this category, the existence of misconceptions in numerous students was true, whereas in the lying posture category, although there were not many correct answers, the wrong answers were not the ones most often selected by the participants, which indicates that there was insufficient knowledge but not a concentration of misconceptions. By analyzing these results in depth, it can be pointed out that the most important error in the category of anatomical knowledge occurred in the item concerning the number of curves of the spine (Table S1, variable V2). Further, 56.21% of students thought that the spine had two curves, which is an indicator of erroneous knowledge of the spine. Although this study did not investigate, through qualitative methodologies, the way in which students explain and understand the spine, an explanation can be based on the theory of reassumption [24] as one of the multiple routes to successful learning [25]. This conceptual error regarding the spine can be explained by using a theoretical framework. It appears that students’ perceptions of the spine are influenced by a reductionist perspective of the body, particularly the trunk. In teaching practice, professionals often simplify the body by dividing it into two parts: the upper body (which includes the shoulder girdle and arms) and the lower body (which encompasses the pelvic girdle and legs). PE teachers frequently employ this simplified division in their classes. Consequently, when students incorporate their understanding of the spine as a basic body structure, they may adopt this simplified view, assuming that the spine has two parts and therefore two curves. This process of integrating naïve knowledge with existing or prior knowledge can lead to misconceptions that impede the deep understanding of new and complex concepts and hinder the acquisition of new scientific knowledge [24].

One of the misconceptions revealed in this study relates to a lack of knowledge regarding proper body posture in different everyday situations. The results indicate a significant belief among participants that lying down is the most stressful posture for the spine (Table S4, variable V8), with options such as lying on the side or lying supine being selected by nearly 50% of the sample. Surprisingly, only one-third of the participants were aware that the standing posture was the most stressful for the body [39]. This misconception can be attributed to experiential or naïve knowledge in which individuals typically associate physical fatigue with the need to sit or lie down to rest and alleviate feelings of fatigue. Consequently, the choice of lying down as the most stressful posture seems illogical. The root of the problem likely lies in the lack of understanding of the concept of stress as applied to body structures and the construction of naïve knowledge specific to the adolescents in the sample. It appears that the concept of stress is not associated with fatigue caused by weight bearing but rather with pain or discomfort resulting from prolonged posture holding. Adolescents’ naïve knowledge is likely derived from experiencing back pain in different situations, regardless of the intensity of the perceived strain. Thus, the discomfort felt during extended periods of lying down or getting up after being in a supine position throughout the night is interpreted as stress. This indicates a failure to apply prior knowledge of the weight distribution and the load borne by intervertebral discs in various positions over time. According to Polanyi [21] and Alexander et al. [22], students’ procedural knowledge is not linked to declarative knowledge of the fundamental concept in question, namely the load. Instead, it was connected to personal perceptions and experiences. Consequently, it can be inferred that their experience consolidated this conceptual error.

Two of the most common errors identified in the study were related to the proper use of standing posture (Table S5, variable V9) and sitting posture in front of a desk (Table S6, variable V12). Both questions involved habitual postures, and the answers provided reflected either appropriate or inappropriate use of the concepts of load or weight transmission and spine stability. For the sitting posture question, incorrect answers were chosen most frequently. Approximately 35% of the participants believed that getting up from a chair facing a desk should involve twisting and flexing the spine (Table S6, variable V11). This response highlights the lack of understanding of the actions that pose a risk to trunk stability. Biomechanics textbooks describe the combined action of flexion and rotation as intervertebral shearing, which is highly detrimental to the back, causing instability and tension in the intervertebral discs [40], thus posing a high risk of injury. Another conceptual error related to proper weight transmission was revealed by the fact that nearly 25% of students selected positions of the spine and pelvis in the standing posture, resulting in a deformation of the physiological curvature of the spine (Table S5, variable V9). Positions involving the anteversion or retroversion of the pelvis lead to changes in natural spinal curves, causing inadequate load transmission and an imbalance in the functional activity of the trunk musculature responsible for maintaining trunk stability. These errors can be attributed to the lack of expert knowledge regarding the natural shape of the back and the functioning of its various structures. For example, an accurate understanding of the number of spinal curves and their components reflects expert knowledge. However, this study indicated that anatomical knowledge was the weakest category analyzed in the TAM (score 5.2, Table S13) and had the highest score in the MM (score 3.8, Table S14).

Finally, it is important to comment on the answers that had a low index score in the TAM (−50%) and the MM (−10%), namely the items about how to carry a backpack on one’s back (Table S8, variable V16) and what the best sleeping posture is (Table S12, variable V23). In both items, the same pattern was repeated. The answers exhibiting the highest scores in the TAM, together with the answers that did not score in either model, were the ones chosen most often, which shows that there is prior knowledge that is correct or is in the process of being correct. In this sense, it seems that most students are aware that a backpack should be carried using two handles, one on each shoulder; however, it does not seem clear to them whether it should also be worn across the chest. Similarly, students understood that the least-harmful positions for resting while lying down are prone and lateral decubitus. There do not seem to be any major conceptual errors in the use of these postures or actions, so it is evident that they lack expert knowledge on these issues.

### 4.3. Contradictions in the Assessed Knowledge

Some of the obtained results seem contradictory and are not due to inattention or memory failure [41]. Students know the names of the four parts of the spine but do not know how many curves they have. This suggests fragmented learning, in which parts are memorized without connecting them to shape changes. Learning about the human body requires an understanding of the association between the curves and the names of the parts of the spine. Additionally, the functions of the spine and trunk muscles are known, whereas aspects of their morphology and surrounding muscles are unknown. Understanding the concept of trunk stabilization and the role of the trunk musculature is complex and important for daily life and PA. The use of body postures and the development of trunk musculature are key elements in promoting a healthy back [37].

### 4.4. Limitations of the Study and Future Lines of Work

Although this study provided a reliable instrument to determine misconceptions and prior knowledge about back health, its small sample size did not allow us to generalize its results. The use of this instrument in future studies with representative samples could help us understand secondary-school students’ preconceptions and errors in greater depth and allow us to address the effect of back-health knowledge. The main limitation of this study is that it cannot go beyond hypothesizing the reasons for these results based on current knowledge. Future studies should address the problems of prior knowledge, naïve knowledge, and erroneous knowledge in the construction of back-health knowledge by incorporating a qualitative perspective. It is necessary to investigate the rationale behind students’ knowledge regarding the use of their body in everyday life. This paper presents an interpretation based on the knowledge gathered from the study sample. However, it is necessary that future studies collect this information from students’ original sources to analyze and interpret it at its source. In this sense, the analysis presented here can be useful to detect the fundamental concepts related to back health and, in this way, analyze and interpret qualitative information from students’ different types of knowledge. This could provide access to understanding how well-established knowledge or conceptual errors can affect healthy habits.

Finally, we consider that knowledge not only affects learning but also that learning, in turn, can provide explanations for different pathologies. Thus, future studies should analyze knowledge’s potential effects on postural changes and musculoskeletal disorders.

### 4.5. Impact on Teaching

Addressing questions such as how knowledge can affect the use of the body and movement in daily life or in PA will help us justify the need to know how to incorporate this knowledge into our teaching practice. The results of this study will not answer these questions directly; however, they will lead us toward an effective approach to teaching about back health. Therefore, these results show prior declarative knowledge related to the naming and location of the parts that will allow us to refer to knowledge such as the alignment of the body axis in relation to the physiological curves of the spine, the control of the anteversion and retroversion of the pelvis in the alignment of the body axis, the spine and musculature of the trunk in the transmission of a load, and the neuromuscular mechanisms for trunk stabilization. Procedural knowledge (such as the use of the body in different postures), maintained postures, at-risk actions (such as combined flexion and rotation movements), and the support, mobilization, and transport of loads are also highlighted. These are all aspects that must be considered when assessing the necessary prior knowledge [42] to distinguish between expert and novice learners [36], as well as understand and assess naïve and misconceived knowledge [24].

These are necessary steps to applying one of the most accepted theories in the field of cognitive psychology—the theory of cognitive change—considering that the possible routes of conceptual change are numerous [25]. Studies on knowledge in science argue that naïve knowledge, misconceptions, and cognitive contradictions or conflicts in knowledge management are at the heart of knowledge acquisition [43,44], and that it is from the awareness that there are other explanations or interpretations of the same phenomenon that we can begin to build the necessary conceptual change. Some studies on the concept of energy have deepened the analysis of knowledge in PE by applying the theory of change [31].

## 5. Conclusions

In conclusion, the HEBACKNOW is a reliable instrument for collecting information on secondary school students’ declarative back-care knowledge in daily life activities to detect prior knowledge and misconceptions.

Although the participants’ declarative knowledge score regarding back health in activities of daily living was moderate, it did not reach the expected level. Conversely, the score for conceptual errors was >20%. The items concerning the location of the spine, the names of different parts of the spine, the function of the spine in the body and trunk musculature, and how to carry weight in bags exhibited the highest scores. Anatomical knowledge related to the number of back curves, the most stressful posture for the back, correct sitting posture in front of a desk, and use of a standing posture were the most common misconceptions.

Although this knowledge’s effect on the acquisition of new back-health knowledge and habits remains to be investigated, the present study provides some explanations for this problem. It is necessary to deepen research into how previous knowledge and conceptual errors can affect the understanding of complex concepts necessary for the proper use of the body and body posture in daily life, such as trunk stability or the transmission of loads in body postures.

## Figures and Tables

**Table 1:** Percentage of scores in models TAM and MM by category and item.

Category	Items	Question	TAM ^1^ %	MM ^2^ %
An	V1	The spine is in…	95.86	2.36
An	V2	How many curves does the spine have?	8.88	56.21
An	V3	What are the different parts of the spine called?	82.84	7.10
An-Fun	V4	The spine has curves in order to…	76.92	1.78
An-Fun	V5	What is the function of the spine in the body?	86.39	4.73
An	V6	Which of the following muscles is a trunk muscle?	20.71	11.24
An-Fun	V7	The function of the trunk musculature is…	88.76	6.51
Stand-P	V8	The most stressful posture for your back is…	26.04	49.71
Stand-P	V9	Which of the following postures is the most adequate?	69.23	24.85
Stand-P	V10	When standing for a while without moving, I should…	75.15	8.28
Sit-P	V11	When sitting for a long time (watching TV, studying)…	75.15	10.65
Sit-P	V12	When sitting at a desk…	29.59	34.91
Sit-P	V13	When sitting at a desk with a computer…	77.51	2.9
BackP-L	V14	When carrying books or large objects a long way, the best option is…	62.13	7.70
BackP-L	V15	When carrying a schoolbag with books, the weight should be…	54.44	15.98
BackP-L	V16	When carrying weight in my schoolbag, I should wear it on…	47.34	3.56
BackP-L	V17	When carrying weight in my schoolbag…	57.99	27.40
BackP-L	V18	When carrying weight in bags…	84.02	11.24
Weight-L	V19	When carrying a heavy load, it is better…	51.48	18.35
Weight-L	V20	When holding heavy loads in your arms, it is better…	57.99	34.91
Weight-L	V21	When lifting heavy objects off the floor…	73.37	18.34
Weight-L	V22	When reaching for an object that is over my head…	77.51	7.10
Lying-P	V23	When sleeping, the best posture is…	49.11	16.57
Lying-P	V24	The surface I sleep on should be…	71.60	14.79

^1^ True answer model (TAM); ^2^ misconception model (MM). Questionnaire categories: anatomical (An), anatomical functional (An-Fun), standing posture (Stand-P), sitting posture (Sit-P), backpack loading (BackP-L), weight load (Weight-L), and lying posture (Lying-P).

## Data Availability

Data Availability Statements are available in https://osf.io/ejdt5/files/osfstorage/6696f39e5a51c406cfe80b74 (accessed on 12 August 2024).

## Supplementary Materials

The following supporting information can be downloaded at: https://www.mdpi.com/article/10.3390/children11080997/s1, Tables S1–S12 show the frequency of answers given by the participants for each variable (V1–V24), each time (T1, T2), and the scoring model application. The procedure for studying the reliability (correlation between measures, Figures S1 and S2; test–retest and Bland–Altman plot, Figures S3–S6) and score results of both models (Tables S13 and S14).
