# Supplementary material for: Back-Health Knowledge and Misconceptions Related to the Daily Life Activities of Secondary School Students
Source: Children (Basel). 2024 Aug 15;11(8):997. doi: 10.3390/children11080997 (PMC11352296; doi:10.3390/children11080997)
Supplement: Supplementary file 1 [file children-11-00997-s001.zip › Supplementary File S1.pdf]

Supplementary File S1

Scoring models

In Tables S1–S12, we can see the answers given by the participants in each variable and timepoint (T1, T2), so we can determine changes in the answers across time. It also shows how the true answer model and misconceptions model were applied.

Table S1: Frequency of responses for the two rounds (T1 and T2), variables 1 (V1) and 2 (V2).

v1 The spine is located in…:
- The abdomen .... a
- The trunk .... b
- The legs c
- The head .... d

Response	T1 (%)	T2 (%)
**a****	3 (1.78)	6 (3.55)
**b*****	162 (95,86)	158 (93.49)
**c***	2 (1.18)	2 (1.18)
**d***	2 (1.18)	3 (1.78)

v2 How many curves does the spine have?
-Two .... a
-Three .... b
- Four c
- I do not know .... d

Response	T1 (%)	T2 (%)
a*	95 (56.21)	86 (50.89)
b**	47 (27.81)	54 (31.95)
c***	15 (8.88)	21 (12.43)
d**	12 (7.10)	8 (4.73)

* Wrong answer (1 point: misconceptions model [MM]; 0 points: true answer model [TAM]). ** Ambiguous answer or "I do not know" (0 points: TAM, MM). *** True answer (1 point: TAM; 0 points: MM).

Table S2: Frequency of responses for the two rounds: variables 3 (V3) and 4 (V4).

v3 How are the different parts of the spine called?
- High, middle, and low .... a
- Neck, back, kidneys, and sacrum .... b
- Cervical, thoracic, lumbar, and sacrum c
- Head, back, and hip .... d

Response	T1 (%)	T2 (%)
a*	12 (7.10)	14 (8.28)
b**	8 (4.73)	6 (3.55)
c***	140 (82.84)	139 (82.25)
d**	9 (5.33)	10 (5.92)

v4 The spine has got curves in order to…
- be wider in its upper side and narrower in its lower side .... a
- support the arms and the legs .... b
- bear a larger amount of weight c
- provide a better support when sitting on a chair .... d

Response	T1 (%)	T2 (%)
a*	3 (1.78)	8 (4.73)
b**	17 (10.06)	26 (15.38)
c***	130 (76.92)	120 (71.01)
d**	19 (11.24)	15 (8.88)

* Wrong answer (1 point: MM; 0 points: TAM). ** Ambiguous answer or "I do not know" (0 points: TAM, MM). *** True answer (1 point: TAM; 0 points: MM).

Table S3: Frequency of responses for the two rounds (T1 and T2): variables 5 (V5) and 6 (V6).

v5 What is the function of the spine in the body?

- To straighten your back and stretch your ligaments to become taller...... a
- To protect the spinal cord, transmit the weight to the lower limbs and provide back flexibility...... b
- To support the viscera and help us breathe...... c
- To flatten the back...... d

Response	T1 (%)	T2 (%)
a*	8 (4.73)	11 (6.51)
b***	146 (86.39)	146 (86.39)
c**	4 (2.37)	6 (3.55)
d**	11 (6.51)	6 (3.55)

v6 Which of the following muscles is a trunk muscle?

- The quadriceps...... a
- The biceps brachii...... b
- The sternocleidomastoid...... c
- The rectus abdominis d

Response	T1 (%)	T2 (%)
a**	16 (9.47)	17 (10.06)
b*	19 (11.24)	22 (13.02)
c**	35 (20.71)	43 (25.44)
d***	99 (58.58)	87 (51.48)

* Wrong answer (1 point: MM; 0 points: TAM). ** Ambiguous answer or "I do not know" (0 points: TAM, MM). *** True answer (1 point: TAM; 0 points: MM).

Table S4: Frequency of responses for the two rounds (P1 and P2): variables 7 (V7) and 8 (V8).

v7 The function of the trunk musculature is:

- To control back stability and support the viscera...... a
- To support fat from the abdomen and the waist...... b
- To embellish the body...... c
- To get a flatter abdomen...... d

Response	T1 (%)	T2 (%)
a***	150 (88.76)	145 (85.80)
b**	8 (4.73)	14 (8.28)
c*	6 (3.55)	6 (3.55)
d*	5 (2.96)	4 (2.37)

v8 The most stressful posture for your back is:

- Lying on one side...... a
- Sitting...... b
- Standing...... c
- Lying face up...... d

Response	T1 (%)	T2 (%)
a*	50 (29.59)	41 (24.26)
b**	41 (24.26)	30 (17.75)
c***	44 (26.04)	62 (36.69)
d*	34 (20.12)	36 (21.30)

* Wrong answer (1 point: MM); 0 points: TAM). ** Ambiguous answer or "I do not know" (0 points TAM, MM). *** True answer (1 point: TAM; 0 points: MM).

Table S5: Frequency of responses for the two rounds (T1 and T2): variables 9 (V9) and 10 (V10).

v9 Which of the following postures is the most adequate?

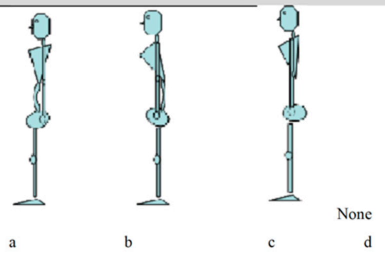

Response	T1 (%)	T2 (%)
a*	34 (20.12)	21 (12.43)
b*	8 (4.73)	8 (4.73)
c***	117 (69.23)	127 (75.15)
d**	10 (5.92)	13 (7.69)

v10 When standing for a while without moving, I should:
- Shift the weight from one leg to the other, trying not to bend or twist the trunk for a long time .... a
- Keep my feet still and twist my neck and trunk to hold conversations .... b
- Wear high-heeled shoes to relax the trunk muscles c
- Remain as steady as possible .... d

Response	T1 (%)	T2 (%)
a***	127 (75.15)	135 (79.88)
b*	14 (8.28)	11 (6.51)
c**	4 (2.37)	6 (3.55)
d**	24 (14.20)	17 (10.06)

* Wrong answer (1 point: MM; 0 points: TAM). ** Ambiguous answer or "I do not know" (0 points: TAM, MM). *** True answer (1 point: TAM; 0 points: MM).

Table S6: Frequency of responses for two rounds (T1 and T2): variables 11 (V11), and 12 (V12).

v11 When sitting for a long time (watching TV, studying, working, etc.), I should:
- put my upper back on the chair backrest and sit on the seat edge .... a
- put my lower back on the chair backrest, keeping the trunk in an upright position and both feet flat on the floor, avoiding thigh pressure. ... b
- turn the spine to talk to someone or fetch something which is on one side or behind me. c
- keep my legs close together and sit with decorum .... d

Response	T1 (%)	T2 (%)
a*	18 (10.65)	28 (16.57)
b***	127 (75.15)	120 (71.01)
c**	4 (2.37)	6 (3.55)
d**	20 (11.83)	15 (8.88)

v12 When sitting by a desk...
- I have to twist and bend my trunk to stand up from the chair .... a
- The desk height should be just below my chest height and I should not rest my forearms and/or elbows on it. .... b
- The bookstand to hold my books and notes when reading is harmful to my neck c
- I should always rest my elbows on the desk .... d

Response	T1 (%)	T2 (%)
a*	59 (34.91)	65 (38.46)
b***	50 (29.59)	61 (36.09)
c**	22 (13.02)	20 (11.83)
d**	38 (22.49)	23 (13.61)

* Wrong answer (1 point: MM; 0 points: TAM). ** Ambiguous answer or "I do not know" (0 points TAM, MM). *** True answer (1 point: TAM; 0 points: MM).

Table S7: Frequency of responses for the two rounds (T1 and T2): variables 13 (V13) and 14 (V14).

v13 When sitting by a desk with a computer
- The screen should be on one side and I have to twist my head to look at it
- The computer keyboard and the mouse should be at my elbows height
- I should sit near the screen to see it better
- I should rest my wrists on it to type on the keyboard

Response	T1 (%)	T2 (%)
a*	5 (2.96)	11 (68.51)
b***	131 (77.51)	124 (73.37)
c**	7 (4.14)	6 (3.55)
d**	26 (15.38)	28 (16.57)

v14 When carrying books or objects some distance away, the best option is to use:
-A handbag ... a
-A backpack ... b
-A shoulder bag ... c
-A rolling backpack ... d

Response	T1 (%)	T2 (%)
a*	10 (5.92)	11 (6.51)
b**	51 (30.18)	61 (36.09)
c*	3 (1.78)	6 (3.55)
d***	105 (62.13)	91 (53.85)

** Wrong answer (1 point: MM; 0 points: TAM). ** Ambiguous answer or "I do not know" (0 points: TAM, MM). *** True answer (1 point: TAM; 0 points: MM).*

Table S8: Frequency of responses for the two rounds (T1 and T2): variables 15 (V15) and 16 (V16).

v15 When carrying a schoolbag with books, the weight should be:
-The least amount of weight possible and never more than 10% of my bodyweight ... a
-As much as I can support without suffering backache and never more than one half of my bodyweight ... b
-It is not important the weight I carry ... c
-I should not support more than a quarter of my bodyweight ... d

Response	T1 (%)	T2 (%)
a***	92 (54.44)	88 (52.07)
b*	23 (13.61)	29 (17.16)
c*	4 (2.37)	7 (4.14)
d**	50 (29.59)	45 (26.63)

v16 When carrying weight in my schoolbag, I should wear it on my back:
- with one strap, worn over one shoulder ... a
- with two straps, worn over one shoulder ... b
- with one strap worn over one shoulder and wound around the chest ... c
- with two straps, each one worn over one shoulder and tightened at the back by a waist strap ... d

Response	T1 (%)	T2 (%)
a*	3 (1.78)	7 (4.14)
b**	83 (49.11)	70 (41.42)
c*	3 (1.78)	7 (4.14)
d***	80 (47.34)	85 (50.30)

* Wrong answer (1 point: MM; 0 points: TAM). ** Ambiguous answer or "I do not know" (0 points TAM, MM). *** True answer (1 point: TAM; 0 points: MM).

Table S9: Frequency of responses for the two rounds (T1 and T2): variables 17 (V17) and 18 (V18).

v17 When carrying weight in my schoolbag:
- It is not important to distribute weight inside the bag properly................ a
- I should pack the heaviest items in the bag close to my body and the lightest ones away from it. b
- I should pack the lightest items in the bag close to my body and the heaviest ones away from it.......................... c
- I should put the heaviest items in the middle of the bag.................................. d

Response	T1 (%)	T2 (%)
a*	16 (9.47)	15 (8.88)
b***	98 (57.99)	102 (60.36)
c*	32 (18.93)	33 (19.53)
d**	23 (13.61)	19 (11.24)

v18 When carrying weight in bags, I should:
- distribute the weight evenly between the two arms .... a
- use my dominant arm for the heaviest weight and my non-dominant arm for the lightest weight .... b
- only use my dominant arm................................ c
- simultaneously use my two arms to hold the weight.. d

Response	T1 (%)	T2 (%)
a***	142 (84.02)	141 (83.43)
b*	14 (8.28)	14 (8.28)
c*	5 (2.96)	7 (4.14)
d**	8 (4.73)	7 (4.14)

* Wrong answer (1 point: MM; 0 points: TAM). ** Ambiguous answer or "I do not know" (0 points: TAM, MM). *** True answer (1 point: TAM; 0 points: MM).

Table S10: Frequency of responses for the two rounds (T1 and T2): variables 19 (V19) and 20 (V20).

v19 When carrying a heavy load, it is better to: (v 1)
-pull a rolling bag.. a
-push a rolling bag................................ b
-carry it on your arms.. c
-distribute them in both hands using two bags.................................. d

Response	T1 (%)	T2 (%)
a**	51 (30.18)	62 (36.69)
b***	87 (51.48)	65 (38.46)
c*	4 (2.37)	12 (7.10)
d*	27 (15.98)	30 (17.75)

v20 When holding heavy loads in your arms, it is better to:
-stretch your arms and keep the object as far as possible from your body........
-stretch your arms and keep the object slightly far from your body...............
-carry the object on your side using only one arm...
-hold the object with both arms to keep it as close as possible to your body....

Response	T1 (%)	T2 (%)
a*	13 (7.69)	16 (9.47)
b*	46 (27.22)	42 (24.85)
c**	12 (7.10)	10 (5.92)
d***	98 (57.99)	101 (59.76)

* Wrong answer (1 point: MM; 0 points: TAM). ** Ambiguous answer or "I do not know" (0 points: TAM, MM). *** True answer (1 point: TAM; 0 points: MM).

Table S11: Frequency of responses for two rounds (T1 and T2): variables 21 (V21), and 22 (V22).

v21 When lifting heavy objects off of the floor, I should:
- keep my feet as far as possible from the object.................. a
- keep my back straight and apply force with my legs.............. b
-keep my legs stretched and apply force with my trunk........ c
-put the object beside my body and lift, twisting and bending my trunk to apply force .... d

Response	T1 (%)	T2 (%)
a*	14 (8.28)	10 (5.92)
b***	124 (73.37)	122 (72.19)
c*	17 (10.06)	16 (9.47)
d**	14 (8.28)	21 (12.43)

v22 When reaching for an object which is over my head, it is better to:
- stretch the arms and the trunk as much as necessary .... a
- climb a ladder to face the object.................. b
- jump to fetch it c
- stand on my tiptoes and stretch my body.. d

Response	T1 (%)	T2 (%)
a**	26 (15.38)	30 (18.34)
b***	131 (77.51)	127 (75.15)
c*	5 (2.96)	6 (3.55)
d*	7 (4.14)	5 (2.96)

* Wrong answer (1 point: MM; 0 points: TAM). ** Ambiguous answer or "I do not know" (0 points: TAM, MM). *** True answer (1 point: TAM; 0 points: MM).

Table S12: Frequency of responses for the two rounds (T1 and T2): variables 23 (V23) and 24 (V24).

v23 When sleeping, the best posture is:
- Face-down.......... a
- Face-up.......... b
- On one side.......... c
- Any one.......... d

Response	T1 (%)	T2 (%)
a*	12 (7.10)	11 (6.51)
b***	83 (49.11)	79 (46.75)
c**	58 (34.32)	64 (37.87)
d*	16 (9.47)	15 (8.88)

v24 The surface where I sleep on should be:
- Firm.......... a
- Soft.......... b
- Firm but comfortable.......... c
- Neither firm nor soft. .... d

Response	T1 (%)	T2 (%)
a*	12 (7.10)	17 (10.06)
b*	13 (7.69)	2 (1.18)
c***	121 (71.60)	121 (71.60)
d**	23 (13.61)	29 (17.16)

* Wrong answer (1 point: MM; 0 points: TAM). ** Ambiguous answer or "I do not know" (0 points: TAM, MM). *** True answer (1 point: TAM; 0 points: MM).
