# Supplementary material for: Back-Health Knowledge and Misconceptions Related to the Daily Life Activities of Secondary School Students
Source: Children (Basel). 2024 Aug 15;11(8):997. doi: 10.3390/children11080997 (PMC11352296; doi:10.3390/children11080997)
Supplement: Supplementary file 1 [file children-11-00997-s001.zip › Supplementary File S2.pdf]

Supplementary File S2

Reliability statistical analysis

The questionnaire was completed twice, with a three-week interval between sessions. To determine the degree of the relationship between the two measurements (T1 and T2) in each model, the means of both runs were plotted on a graph, while the regression lines of the points (slope, intercept, and R2) and their correlation coefficients (r) were calculated.

Standard deviations of means and differences, intraclass correlation coefficients (ICCs) based on two-way mixed-effects models of multiple measure means with absolute agreement [44] and 95% confidence intervals (CI), standard error of measurement (SEM), minimum detectable change (MDC), and coefficient of repeatability (CR) were calculated. A confidence level of 95% was set for the minimum detectable change, corresponding to a z-value of 1.96. ICCs < 0.40, 0.40–0.75, and > 0.75 represented poor, moderate, and excellent agreement, respectively.

To determine whether the differences between the two measurements in each model yielded significantly different values, an independent sample t-test was performed, considering this new variable as the difference between the scores at T1 and T2.

The error and range of agreement of the measurements were calculated to determine the relationship between the measurement error and hypothetical true value. This was performed using a Bland-Altman plot. The error was interpreted as significant if the line of equality between the measurements was not within the CI of the mean of the differences between the measurements. For this criterion, the requirement that the difference between the measurements of the same model should not exceed 5% of the total value of the measurement scale was added. Thus, a difference > 0.5 points on a maximum scale of 10 was not accepted. The association between the differences and overall mean scores was calculated using regression analysis.

To more clearly visualize how the errors in the scores of the different models varied, the Bland-Altman plot was plotted as a function of the variability of the proportional differences. Therefore, the quotients of the differences, means of each contrasting model, and their percentages were calculated. Using these values, the median variance, 95% confidence intervals, and agreement intervals were recalculated.

To analyze the floor/ceiling effect, the highest (9–10 points) and lowest (0–1 point) response percentages at T1 were calculated. A floor/ceiling effect in the responses was considered to exist when more than 15% of the participants provided extreme responses.

To analyze the discriminatory capacity of the scores obtained by the participants in the questionnaire, the total means of the first pass were used, which were divided into four groups by quartiles, and a one-factor ANOVA was applied.

Statistics correlation

Plotting the mean scores of both administrations and calculating the slope (MTA, m = 0.69; MM, m = 0.73) of their linear function showed a positive relationship between the measurements at different times in the two models (T1 and T2). Thus, participants' low T1 scores were accompanied by low T2 scores, and high T1 scores led to high T2 scores (Figures S1 and S2).

The correlation of the items with the line showed a low-to-moderate relationship between the measurements (MTA, $R^2 = 0.36$; MM, $R^2 = 0.40$). The correlation coefficients between the scores were good and significant (MTA: $r = 0.60$; MM: $r = 0.64$; $p < 0.001$).

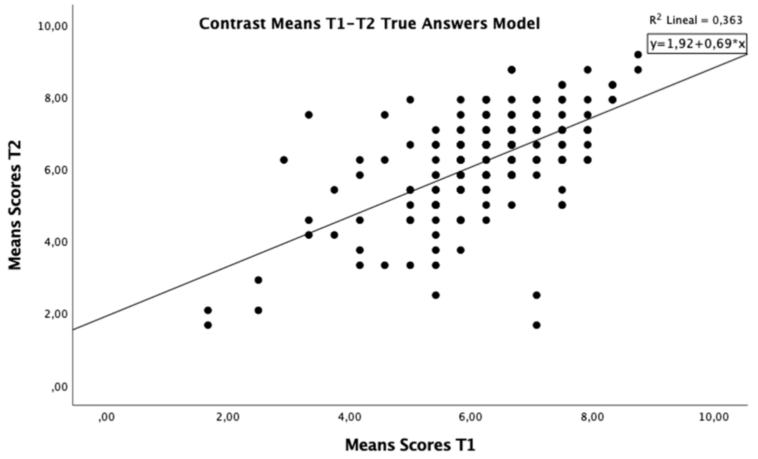

Figure S1

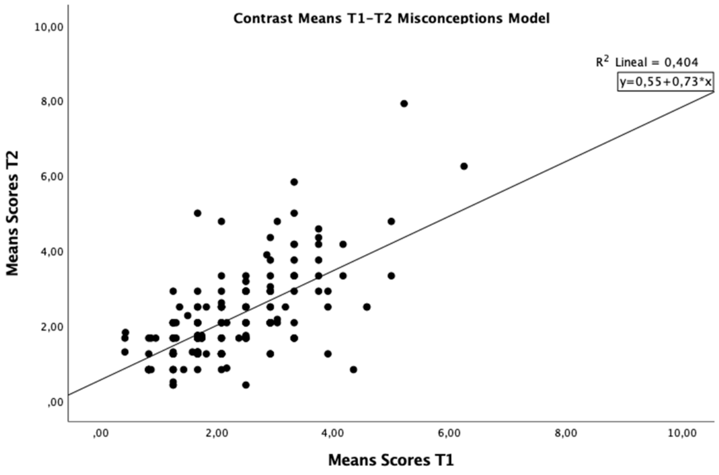

Figure S2

Figures S1–S2.: Representation of the T1 and T2 scores in the scoring models (TAM, MM) and the linear function expressing the relationship between the data sets at the different time points (T1 and T2).

Test-retest

The test-retest results for the measurement models for both total and category scores are shown in Tables S13 and S14.

The ICCs were high for all scores (TAM = 0.75; MM = 0.77). For each of the seven conceptual categories, the ICCs ranged from acceptable to fairly high (TAM; 0.50–0.77 and MM; 0.52–0.67). The SEM (TAM; 0.62–2.12 and MM; 0.45–1.79) and the MDC, (TAM; 1.71–5.87 and MM; 1.26–4.88) were acceptable for all measurements. The difference in means between the test and retest indicated that there were no significant differences in any variable, except for the differences in standing knowledge scores in the TAM ($t_{168} = 3.35$; $p = 0.001$) and MM ($t_{168} = 2.69$; $p < 0.001$). The mean difference was lower than the

SEM and MDC, and the CR was twice as low as the SD for all questionnaire values, except for the standing posture knowledge category.

Table S13: Test-retest repeatability of questionnaire scores on knowledge of back care in activities of daily living. True answers model.

True answers model	M-T1 (SD)	M-T2 (SD)	MD (SD)	ICC	CI (95%)	CR	SEM	MDC	r
**Total**	6.23 (1.28)	6.22 (1.46)	-.01 (1.23)	.75	.66-.81	2.42	0.62	1.71	.60*
**Anatomic**	5.2 (1.6)	5.3 (1.9)	.12 (1.93)	.56	.41-.68	3.77	1.28	3.54	.40*
**Anatomic function**	8.4 (2.5)	8.1 (2.6)	-.30 (2.07)	.50	.29-.61	5.87	2.12	5.87	.31*
**Standing posture**	5.7 (2.6)	6.4 (2.7)	.7 (2.75) *	.64	.51-.73	5.39	1.65	4.57	.47*
**Sitting posture**	6.1 (2.9)	6 (3)	-.06 (3.22)	.57	.42-.68	6.31	2.11	5.86	.40*
**Lying posture**	5.1 (3.1)	5.5 (3.2)	-.37 (2.83)	.77	.68-.83	5.55	1.36	3.76	.62*
**Backpack loading**	6.1 (2.3)	6 (2.7)	.12 (2.40)	.71	.60-.78	4.70	1.29	3.58	.55*
**Weight load**	6.5 (2.5)	6.1 (3)	-.37 (2.58)	.71	.61-.79	5.06	1.39	3.86	.56*

Significance levels * $p < .01$, t-tests for differences between T1 and T2 means, and correlation coefficients. M: mean difference; SD: standard deviation; ICC: intraclass correlation coefficient; CI: confidence interval; CR: coefficient of reproducibility; SEM: standard error of measurement; MDC: minimal detectable change; r: correlation coefficient

Table S14: Test-retest repeatability of questionnaire scores on knowledge of back care in activities of daily living. Misconceptions model.

Misconceptions model	M-T1 (SD)	M-T2 (SD)	MD (SD)	ICC	IC (95%)	CR	SEM	MDC	r
**Total**	2.29 (1.02)	2.22 (1.17)	-.07 (0.95)	.77	.69-.83	1.86	0.45	1.26	.64**
**Anatomic**	3.80 (1.70)	3.52 (1.98)	-.28 (2.11)	.52	.35-.64	4.13	1.46	4.05	.35**
**Anatomic function**	0.81 (1.91)	0.88 (1.92)	.07 (2.09)	.57	.42-.69	4.10	1.37	3.80	.40**
**Standing posture**	3.21 (2.88)	2.61 (2.49)	-.60 (2.91) *	.62	.49-.72	5.70	1.79	4.97	.45**
**Sitting posture**	1.63 (1.41)	1.64 (1.39)	.01 (1.58)	.53	.37-.66	3.10	1.08	3.00	.36**
**Lying posture**	1.57 (2.45)	1.33 (2.53)	.03 (2.72)	.58	.43-.69	5.33	1.76	4.88	.41**
**Backpack loading**	1.34 (1.73)	1.62 (2.04)	-.28 (1.88)	.67	.55-.76	3.69	1.08	3.00	.51**
**Weight load**	1.99 (2.09)	2.03 (2.22)	.03 (2.24)	.63	.50-.71	4.38	1.36	3.77	.46**

Significance levels * $p < .01$, t-tests for differences between T1 and T2 means, and correlation coefficients. M: mean difference; SD: standard deviation; ICC: intraclass correlation coefficient; CI: confidence interval; CR: coefficient of reproducibility; SEM: standard error of measurement; MDC: minimal detectable change; r: correlation coefficient

Bland-Altman plot

The graphical representation (Bland-Altman plot) of the means of the two runs in relation to their differences, both for absolute (Figures S3, S4) and relative (Figures S5, S6) values, and the expression of their linear function TAM ($y = -1.04 + 0.17x$) MM ($y = -0.44 + 0.16x$), showed a tendency for the differences between the means of the two models to increase slightly as their means increased.

The measurement errors for both models were small, in no case exceeding 5%. The limits of agreement of the TAM (2.40%; -2.42%) and MM (-1.49%; 1.79%) scores, calculated from the standard deviation of the difference between the TAM (-0.01) and MM (-0.07) means, showed that 96% of the TAM and MM scores were within an acceptable range of measurement errors. These results indicate that the different model measurements had high levels of agreement or concordance. However, the variance of the MM was higher than that of the TAM.

Analysis of the floor/roof effect showed that it did not occur, as the frequency of scores between 0–1 and 9–10 was 0% for the TAM and 5.9% for the MM. The results of the discriminant capacity of the total mean scores obtained in the application of the questionnaire showed significant differences ($p < 0.01$) between all quartiles in which the variables were grouped.

A study of the effects of age and gender on the scores of the two models indicated that there was no interaction effect (TAM: $F_{(4, 168)} = 1.11$, $p = 0.35$, $\eta^2 = 0.03$; MM: $F_{(4, 168)} = 1.93$, $p = 0.11$, $\eta^2 = 0.05$) and no main effect of age (TAM: $F_{(4, 168)} = 1.53$, $p = 0.45$, $\eta^2 = 0.03$; MM: $F_{(4, 168)} = 1.56$, $p = 0.18$, $\eta^2 = 0.04$). Gender was the only variable that showed significant values in the main effects analysis in both scoring models (TAM: $F_{(4,168)} = 7.20$, $p < 0.01$, $\eta^2 = 0.03$; MM: $F_{(4,168)} = 8.43$, $p < 0.01$, $\eta^2 = 0.05$), although their effect sizes were very small in both models. Post-hoc tests showed that the TAM scores for boys (M 6.50, SD 0.17) were significantly higher ($p < 0.01$) than those for girls (M 5.90, SD 0.16), whereas for MM, the scores for girls (M = 2.63, SD = 0.12) were significantly higher ($p < 0.01$) than those for boys (M 2.11, SD 0.13). This result indicates that neither of these variables influenced the differences in scores in either model.

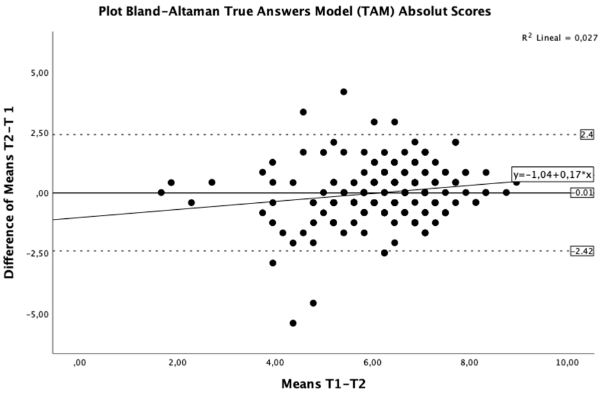

Figure S3

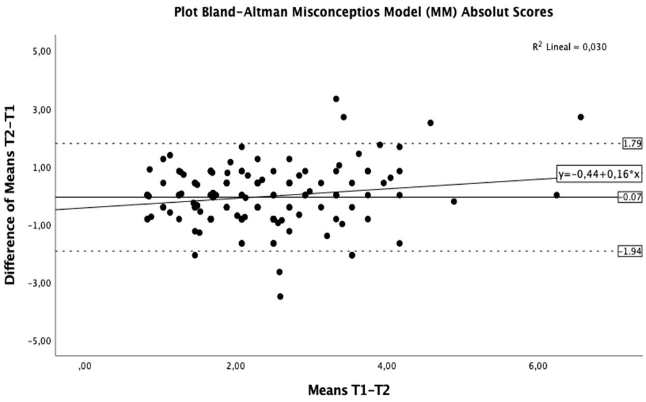

Figure S4

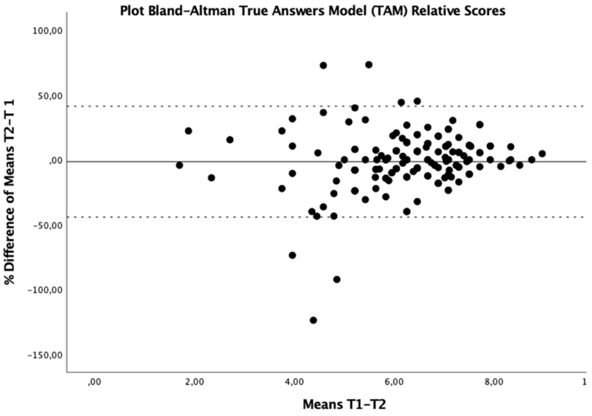

Figure S5

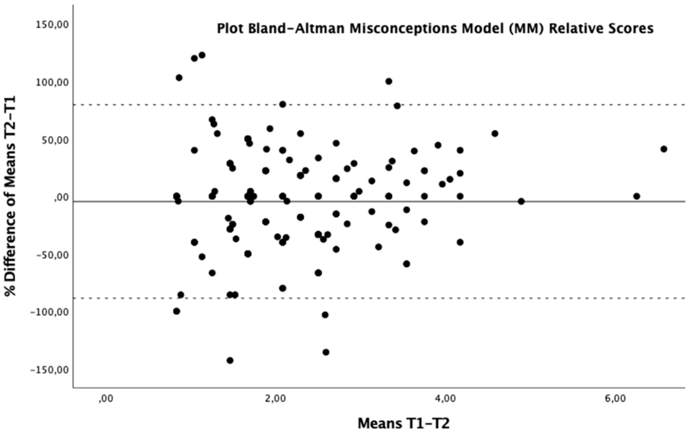

Figure S6

Figures S3, S4, S5, S6: Bland-Altman Plot of the scores obtained in both timepoints of the knowledge questionnaire in absolute and relative values.
